# The First Genomic Analysis of Visna/Maedi Virus Isolates in China

**DOI:** 10.3389/fvets.2022.846634

**Published:** 2022-06-24

**Authors:** Jian-Yong Wu, Xiao-Yun Mi, Xue-Yun Yang, Jie Wei, Xiao-Xiao Meng, Hongduzi Bolati, Yu-Rong Wei

**Affiliations:** Xinjiang Key Laboratory of Animal Infectious Diseases, Institute of Veterinary Medicine, Xinjiang Academy of Animal Science, Ürümqi, China

**Keywords:** Maedi-Visna virus, provirus, Illumina sequencing, cloning, phylogenetic analysis

## Abstract

Visna/Maedi virus (VMV) is a neglected pathogen that damages sheep and goats' nervous and respiratory systems. The virus was discovered 80 years ago and has been endemic in China for nearly four decades; nevertheless, there is little information regarding Chinese isolates' genotypes and genomic characteristics. In this study, the proviral DNA of strains isolated in 1985 and 1994 were extracted, and the proviral DNA was subjected to Illumina sequencing combined with Sanger sequencing of poor coverage regions. The results showed that the two isolates were clustered with genotype A2 and shared 78.3%−89.1% similarity to reference VMV genome sequences, with the highest similarity (88.7%−89.1%) to the USA strain USMARC-200212120-r (accession no. MT993908.1) and lowest similarity (78.3%−78.5%) to the Italian strain SRLV009 (accession no. MG554409.1). A maximum-likelihood tree showed that the Chinese VMV strains and the USA strain 1150 (accession no. MH916859.1) comprise a monophyletic group with a short tree branch. Our data filled the gap in genomic analysis and viral evolution in Chinese VMV strains, and would be benefit China's source-tracing and eradication program development in China.

## Introduction

Maedi-Visna disease is a chronic, wasting disease that affects several mammals in the genera *Capra* and *Ovis* (1). The disease is caused by Visna/Maedi virus (VMV, species *Visna-Maedi virus*), a member of the family Retroviridae, genus *Lentivirus*, which is collectively referred to as small ruminant lentiviruses (SRLVs) together with the Caprine Arthritis-Encephalitis Virus (2). VMV crosses species barriers and has been found in wild ruminants such as red deer, Alpine ibexes, Passirian goats, and Mouflon (3–7). The virus transmits primarily through body fluids such as respiratory exudates, colostrum and milk from infected ewes (1). Vertical transmission through the placenta and semen during pregnancy and mating is an alternative for animal infection with VMV (8–10). The VMV-infected animals manifest continuous and progressive pathological damage to joints, breasts, lungs, and the nervous system (11, 12). In addition, VMV causes lifelong subclinical infections, and only 30% of subclinical VMV infections present clinical symptoms following an incubation period of about 3–4 years (13). The disease causes direct economic losses, including reduced life length, less milk production, and shorter lactation duration (14, 15).

Maedi-Visna disease was initially described in Iceland in 1939 (16). The virus spread to Denmark, Finland, France, Hungary, and Norway through the inter-country trade of breeding sheep between 1960 and 1990 (17). VMV has spread worldwide except for New Zealand and Australia (13, 17). In China, Maedi-Visna disease was identified in 1985 (18), and it was believed to be an infectious disease with individual seroprevalence of 4.6%−50.0% and herd seroprevalence of 100% (19–21). The disease is listed as a Class II animal disease by the Ministry of Agriculture and Rural Affairs of the P.R. China and as a notified terrestrial animal disease by World Organization for Animal Health.

Visna/Maedi virus is an enveloped virus with double-stranded RNA of 8.4–10 kb in length. The nucleic acid encodes three structural protein genes: *env, gag*, and *pol* (22). *Pol* possesses the longest open reading frame, located in the middle of the genome, encoding a pol protein involved in replication and DNA integration (23). The *gag* gene encodes a structural protein comprised of three subunit proteins (capsid, matrix, and nucleocapsid proteins), which stimulates the production of antibodies in the host and protects genomic DNA (24). There are three non-structural protein genes (*rev, tat*, and *vif* ), and in genome 5′ and 3′ ends, a long terminal repeat (LTR) comprises U5. The R and U3 regions are responsible for host tropism for monocytes, macrophages, and dendritic cells (25).

The *gag* and *gag*-*pol* genes and LTR sequences are markers for dividing SRLVs into five genotypes (A–E) (26). Of which, Genotypes A and B are MVV-like and CAEV-like strains that dominated worldwide, respectively, while genotypes C, D and E were only found in Europe, i.e., Group C strains in Norwegian sheep and goats, genotype D strains in sheep and goats in Switzerland and Spain, and genotype E in Italy (27–29). VMV or VMV-like viruses are grouped into genotype A, divided into 22 subgenotypes, including A1–A22 (26, 30). The subgenotype presents a geographical distribution and is clustered into additional subgroups. For example, the USA has only A2 genotype strains to date; however, these are classified further into four subgroups based on a neighbor-joining tree of partial gag sequences (31).

Although VMV was identified in China nearly four decades ago with exceptionally high seroprevalence in Chinese sheep herds, little is known regarding VMV genomic characteristics. This study aimed to characterize two VMV strains isolated in 1985 and 1994 and determine the genotype and the genetic relationship with VMV outside China. We employed a next-generation sequencing method to obtain draft maps of two Chinese isolates. Cloning and Sanger sequencing were performed to obtain precise VMV genome sequences. We also conducted a detailed genomic comparison of Chinese isolates and related genomic sequences reported. The research results will provide a basis for VMV control in China.

## Materials and Methods

### VMV Provirus Genomic DNA Extraction

**Figure 1:**
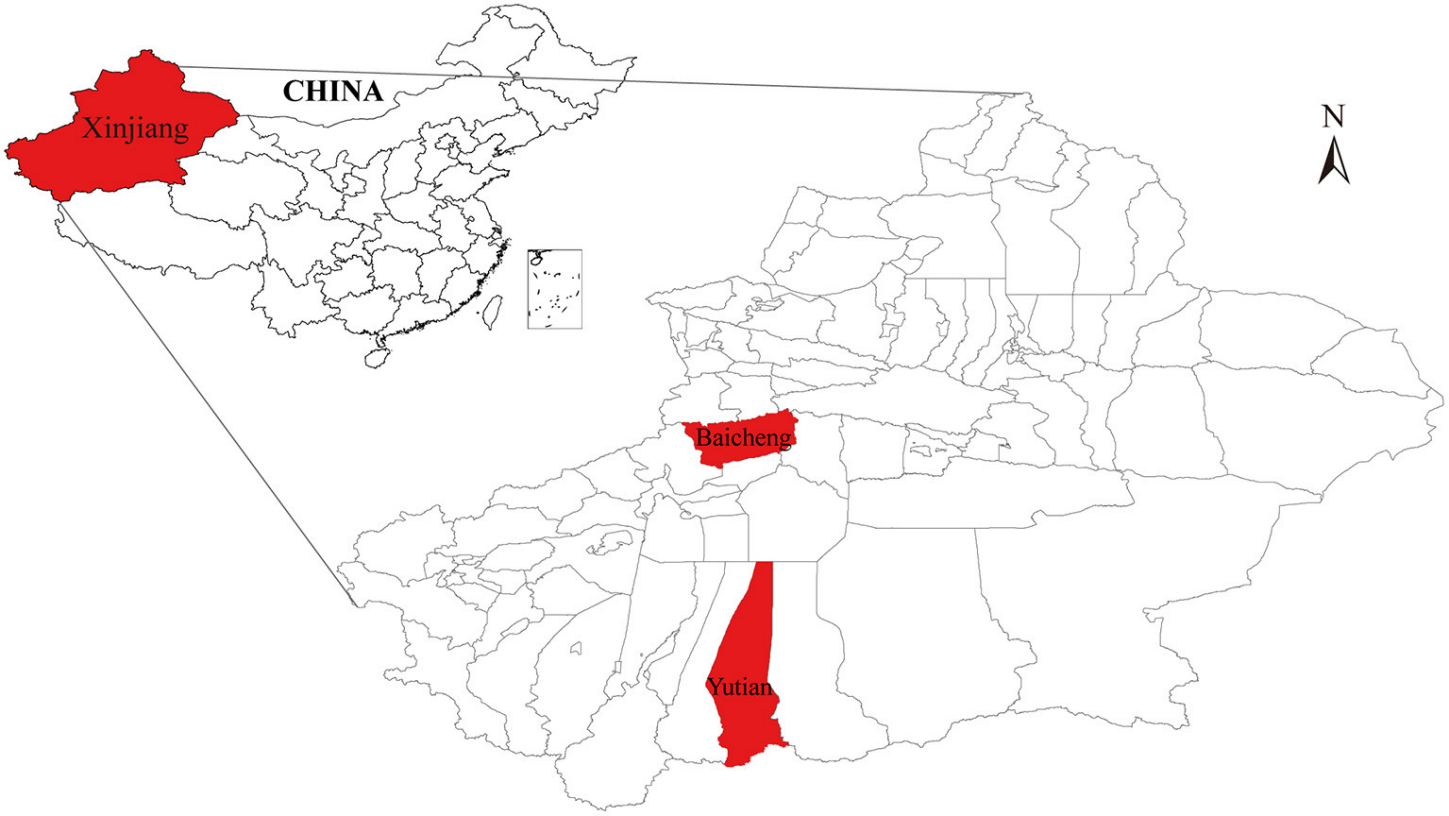
A simplified map of Xinjiang (Northwest China) where the Chinese Visna/Maedi virus strains were isolated. The geographical location of the strains isolated in the study are marked in red.

The two VMV isolates named CMV-1 and XM-MDV30 were isolated from sheep in Baicheng and Yutian Counties in Xinjiang Uygur Autonomous Region, northwest China in 1985 and 1994 (Figure 1) (18, 32), and were stored in liquid nitrogen. Proviral DNA was extracted from 1,000 μl of the cell pellets using a mini-DNA kit (QIAGEN) according to the manufacturer's instructions.

### Illumina Sequencing

These VMV proviral DNA extracts were sent to Novagene Co., Ltd (Nanjing, China) for library construction and metagenome sequencing. Raw reads were processed by Novagene Co., Ltd (Nanjing, China) to remove adapters, filter out reads originating from host sequences, remove chimeras within less than three mismatches and low-quality reads (mean value ≤20) over a certain percentage (the default was 40%). The obtained clean data were assembled using SOAPdenovo2 software (33). Gapclose (version 1.12) software was used to fill the gap in preliminary assembly results and removed the same lane pollution by filtering the reads with low sequencing depth (less than 0.35 of the average depth) to obtain the final assembly result. Fragments below 500 bp were filtered out and the result was counted for gene prediction. The assembled sequences were mapped to VMV strain kv1772 (GenBank no. NC_001452.1) and pinpointed the deletions and insertion sites or fragments. In addition, the clean data were mapped to kv1772 using Geneious prime 2020.0.3 (34) and the sequence coverage and depth were calculated.

### Cloning the Full-Length Genome

Six primer sets (Table 1) were designed based on the assembled sequences of VMV strain CMV-1 using Oligo 7 software to obtain precise sequences of poor coverage regions. The amplified fragments were visualized on 1.5% (w/v) agarose gels in TAE (0.04 M Tris-acetate, 0.001 M EDTA) buffer and purified using a DNA Gel Extraction Kit (Tiangen, China). The purified products were then cloned into the pGEM-T Easy Vector (Promega, Madison, USA) at 16°C for 1 h. Ligated products were transformed into competent DH5α *Escherichia coli* (Tiangen), and recombinant clones were screened on LB/ampicillin/ IPTG/X-Gal plates after incubation at 37°C overnight. We selected and sequenced 3–5 white colonies for each product.

**Table 1:** Primers used for PCR amplification.

**Segment**	**Primer**	**Sequence**	**Tm (**°**C)**	**Length (bp)**
Segment 1	MVV-1F	GACAGAGAACAAATGCCTTCC	55.7	1552
	MVV-1R	TCTTACAGATCTTAATGCGGT		
Segment 2	MVV-2F	GCTAACATGGATCAGGCAAGA	53.5	1748
	MVV-2R	CCTSATCTCCCTCCATTAACTT		
Segment 3	MVV-3F	GCAGAAGTTAGTAGGGGATTT	53.3	1703
	MVV-3R	GAACTCCCGTAGTGTGTTCTA		
Segment 4	MVV-4F	GGTTATGAAATGGTATGCTATGT	54.2	1631
	MVV-4R	CTTCTGCTTACCTTCTGTCAA		
Segment5	MVV-5F	CCGAGCAGAGTGACCTGGAAG	6349	1688
	MVV-5R	GCGCATGTCCATTTATTGCTT		
Segment 6	MVV-6F	CGGAATGCAAAAATGCTACTT	55	1621
	MVV-6R	CAGTTGACTCCTTTATTTCTCCA		

### Similarity Analysis of Whole Genome Sequences

Small ruminant lentiviruses genome sequences were downloaded from GenBank (Table 2), and SRLVs of genotype A and VMV were screened after confirming their species and genotypes. Whole-genome similarity matrixes were then generated for the whole-genome sequences with the known SRLVs genotype A and VMV strains using the Sequence Demarcation Tool v1.2 (35).

**Table 2:** List of Visna/Maedi virus used in the complete genome analyses.

**Strain name**	**Genbank no**.	**Host**	**Year**	**Country**
SA-OMVV	NC_001511.1	*Ovis aries*	1987	South Africa
USMARC-199916193-r	MT993918.1	*Ovis aries*	2006	USA
USMARC-200117502-r	MT993906.1	*Ovis aries*	2006	USA
USMARC-200216049-r	MT993907.1	*Ovis aries*	2005	USA
USMARC-200312088-r	MT993910.1	*Ovis aries*	2007	USA
USMARC-200312013-r	MT993909.1	*Ovis aries*	2007	USA
USMARC-201373037-1	MT993905.1	*Ovis aries*	2017	USA
USMARC-200023230-1	MT993904.1	*Ovis aries*	2006	USA
USMARC-200103342-1	MT993902.1	*Ovis aries*	2006	USA
USMARC-199835918-1	MT993903.1	*Ovis aries*	2007	USA
USMARC-200323455-1	MT993901.1	*Ovis aries*	2007	USA
USMARC-200303332-1	MT993898.1	*Ovis aries*	2006	USA
USMARC-200303038-1	MT993897.1	*Ovis aries*	2006	USA
USMARC-200103515-1	MT993899.1	*Ovis aries*	2006	USA
USMARC-200303013-1	MT993896.1	*Ovis aries*	2006	USA
USMARC-200303013-1	KY358787.1	*Ovis aries*	2006	USA
USMARC-200050064-r	MT993900.1	*Ovis aries*	2006	USA
USMARC-200212120-r	MT993908.1	*Ovis aries*	2006	USA
USMARC-199916128-2	MT993917.1	*Ovis aries*	2006	USA
USMARC-200177363-2	MT993916.1	*Ovis aries*	2006	USA
USMARC-200335185-2	MT993915.1	*Ovis aries*	2007	USA
USMARC-200016283-2	MT993914.1	*Ovis aries*	2006	USA
USMARC-200106929-2	MT993913.1	*Ovis aries*	2004	USA
USMARC-200106932-2	MT993912.1	*Ovis aries*	2006	USA
USMARC-199906011-2	MT993911.1	*Ovis aries*	2003	USA
USMARC-1999060	KY358788.1	*Ovis aries*	2003	USA
1150	MH916859.1	*Ovis aries*	2017	USA
EV1	S51392.1	*Ovis aries*	1991	British
s7631	KT453990.1	*Ovis aries*	2011	Switzerland
s7385	KT453989.1	*Ovis aries*	2011	Switzerland
g6221	KT453988.1	*Ovis aries*	2011	Switzerland
—	AY445885.1	*Capra hircus*	2004	Switzerland
697	HQ848062.1	*Ovis aries*	2005	Spain
P1OLV	AF479638.1	*Ovis aries*	2004	Portugal
Jord1	KT898826.1	*Ovis aries*	2007	Jordan
RLV009	MG554409.1	*Capra hircus*	2017	Italy
SRLV038	MH374287.1	*Ovis aries*	2017	Italy
SRLV_VdA	MH374291.1	*Capra hircus*	2017	Italy
SRLV007	MG554408.1	*Capra hircus*	2017	Italy
SRLV004	MG554405.1	*Capra hircus*	2017	Italy
SRLV003	MG554404.1	*Capra hircus*	2017	Italy
SRLV005	MG554406.1	*Capra hircus*	2017	Italy
SRLV032	MH374286.1	*Capra hircus*	2017	Italy
SRLV024	MH374283.1	*Capra hircus*	2017	Italy
SRLV026	MH374285.1	*Capra hircus*	2017	Italy
SRLV025	MH374284.1	*Capra hircus*	2017	Italy
SRLV006	MG554407.1	*Capra hircus*	2017	Italy
SRLV002	MG554403.1	*Capra hircus*	2017	Italy
kv1772	NC_001452.1	—	—	Iceland
LV1	M10608.1	—	—	Iceland
1514	M60610.1	—	1991	Iceland

—*, the strain name is unknown*.

### Phylogenetic Analyses

The sequences were aligned with sequences of other VMVs (Table 2). Multiple sequence alignment was performed using MAFFT version 7 (https://mafft.cbrc.jp/alignment/server/) (36). The alignment was manually checked and end-trimmed to match the obtained *env, gag, pol, rev, tat*, and *vif* genes, and the conserved regions were obtained after analyzing using Gblocks 0.91b (http://www.phylogeny.fr/one_task.cgi?task_type=gblocks). The substitution saturation of sequences was done using DAMBE 7.0.35. The likelihood tree generated using iqtree-1.6.12 software (http://www.iqtree.org/release/v1.6.12) with GTR + G + I (whole genome sequence, *env, gag, pol* and *vif* ), TVM + G (*rev*), and GTR + G (*tat*). A consensus tree was constructed from the output file produced in the BI analysis using FigTree v. 1.4.4 (http://tree.bio.ed.ac.uk/software/figtree/).

## Results

### Illumina Sequencing

**Figure 2:**
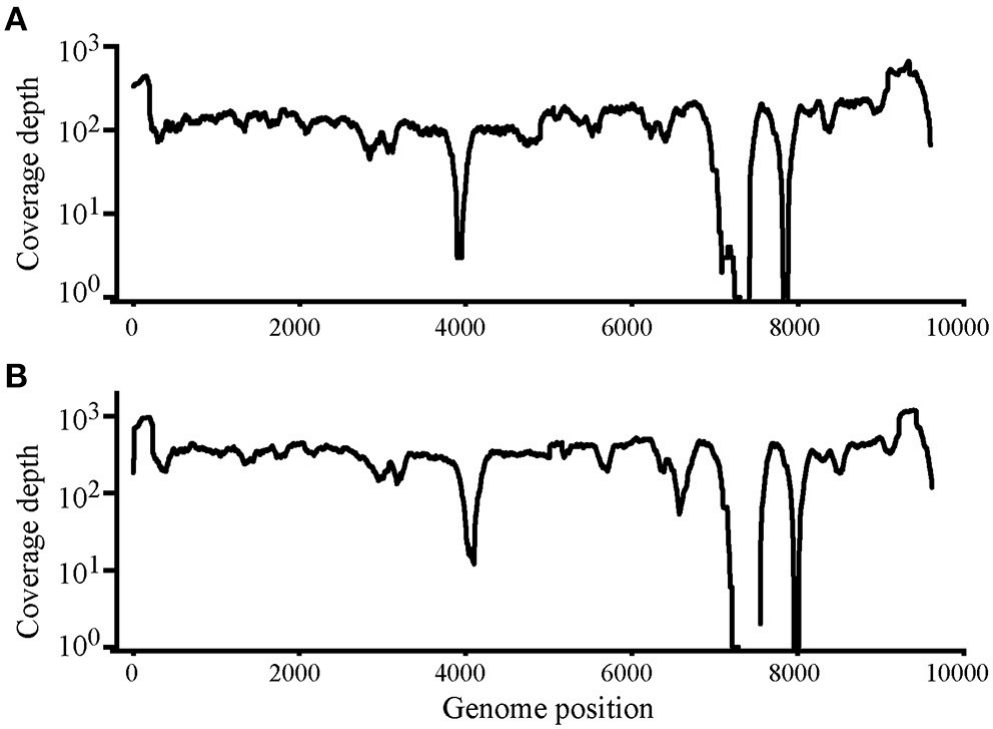
Read depth analysis of Chinese Visna/Maedi virus strains CMV-1 **(A)** and XM-MDV30 **(B)** using Illumina Sequencing reads. Coverage of each position on the VMV genome is indicated on the y axis with a log_10_ scale. The *x*-axis indicates the genome position on the VMV genome.

Using Illumina sequencing, 2,229 and 2,835 Mb of clean data were obtained; clean and raw data percentages were 88.4 and 87.2%, respectively (Supplementary Table S1). The reads were deposited in SRA with BioProject ID PRJNA824058 (https://www.ncbi.nlm.nih.gov/bioproject/PRJNA824058). The analysis showed that nearly complete genome sequences were obtained. Each nucleotide was sequenced more than 100× , except that in genome sites 3,900–4,000 and 7,400–7,900, sequence depth was 100× coverage in most nucleotide sites (Figure 2). Another method was needed to obtain accurate complete genome sequences.

### Genome Cloning and Sanger Sequencing

**Figure 3:**
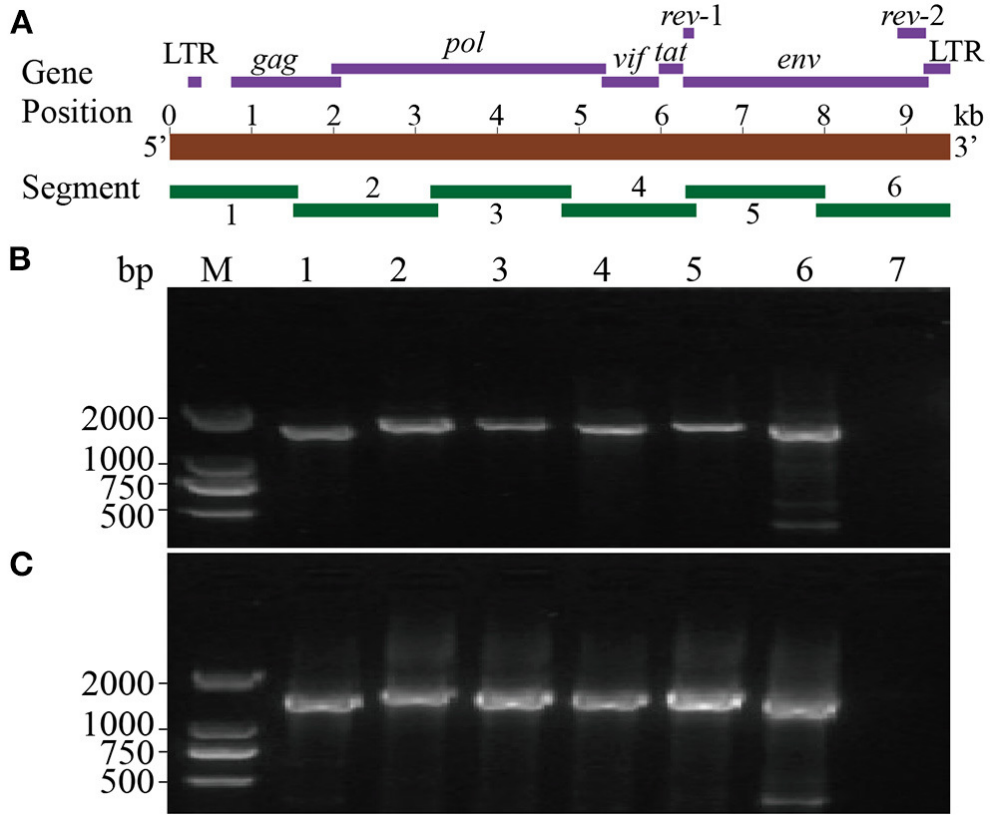
Whole-genome amplification of Chinese Visna/Maedi virus strains. **(A)** Schematic diagram of the VMV genome. Purple bands indicate the position of encoded genes. Red band indicates the length of VMV genome. Green bands indicate the position of six amplified segments. **(B)** Genomic amplification results of six segments of VMV strain CMV-1. **(C)** Genomic amplification results of six segments of VMV strain XM-MDV30. M, DNA marker; 1, segment 1; 2, segment 2; 3, segment 3; 4, segment 4; 5, segment 5; 6, segment 6; 7, blank control.

Six segments (segments 1–6) were amplified within lengths of about 1,600, 1,800, 1,700, 1,600, 1,700, and 1,600 bp (Figure 3), respectively. The amplified segments were then cloned and sequenced using Sanger sequencing. The obtained sequences were assembled with DNAMAN 8.0 with lengths of 9,171 and 9,170 bp in CMV-1 and XD-MDV30, respectively. The genomes were annotated using Geneious prime 2020.0.3 and deposited in GenBank (Accession nos. MZ313871 and MZ313872) with strain designations of CMV-1 and XM-MDV30, respectively.

### Genotype Identification

**Figure 4:**
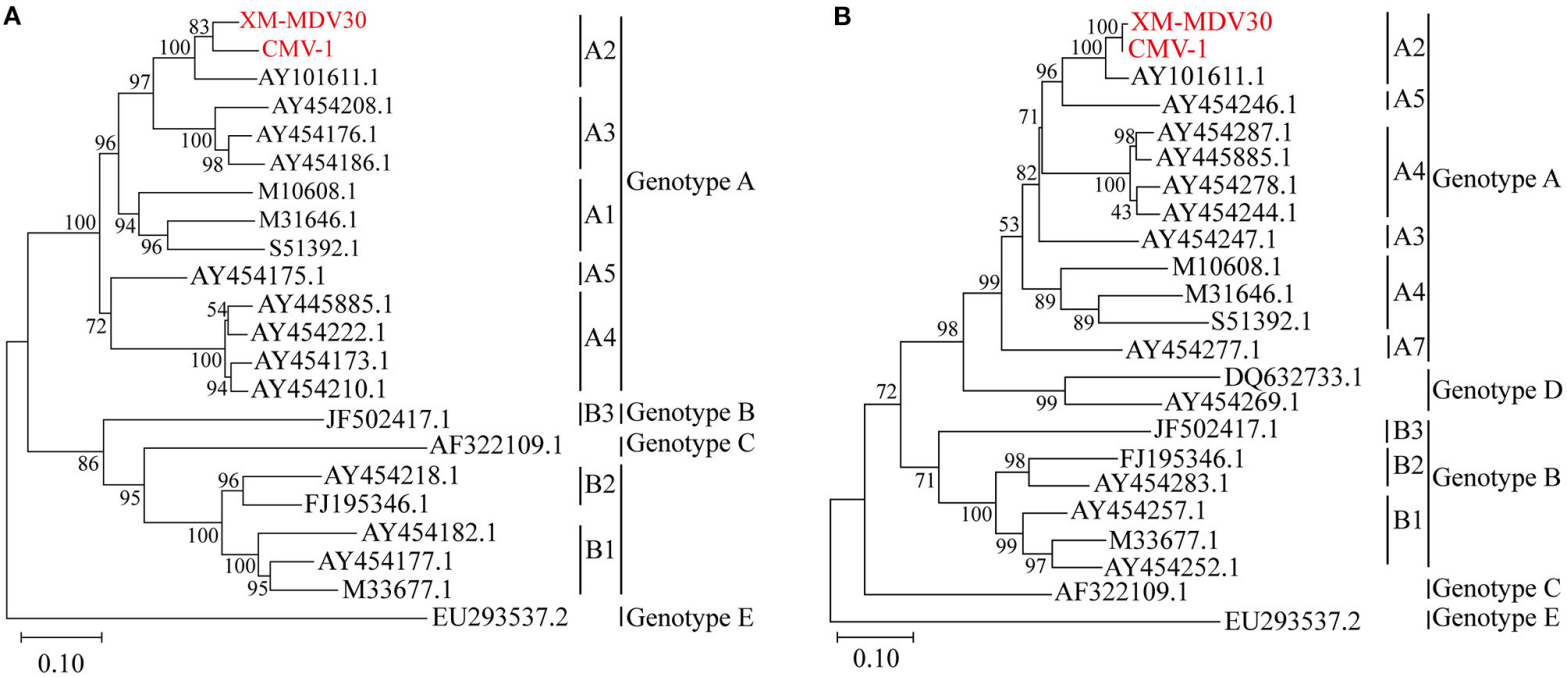
Genotyping of Visna/Maedi virus isolates. The evolutionary history was inferred using the maximum likelihood method based on the Kimura 2-parameter model (37). **(A)** Maximum-likelihood tree based on a 684-bp fragment of the partial gag fragment with 1,000 bootstrap replicates. **(B)** Maximum likelihood tree based on 1,133 bp long fragment of the 1.2 kb gag-pol fragment with 1,000 bootstrap replicates. The VMV genomes sequenced are marked in red. The scale bar indicates the number of nucleic acid changes per site.

After sequencing, two genotyping methods were selected based on the partial *gag* gene (684 bp) and *gag*-*pol* gene (1.8 kb). Gene sequences of several closely related SRLV species were downloaded from GenBank. Phylogenetic analysis was performed to align these two sequences with reference sequences (Figures 4A,B). The result showed that Chinese strains CMV-1 and XM-MDV30 were grouped into the SRLV genotype A and further divided into subgenotype A2 branch based on the phylogenetic tree, indicating that two of the earliest Chinese VMV strain had only one subgenotype.

### Similarity Analysis

**Figure 5:**
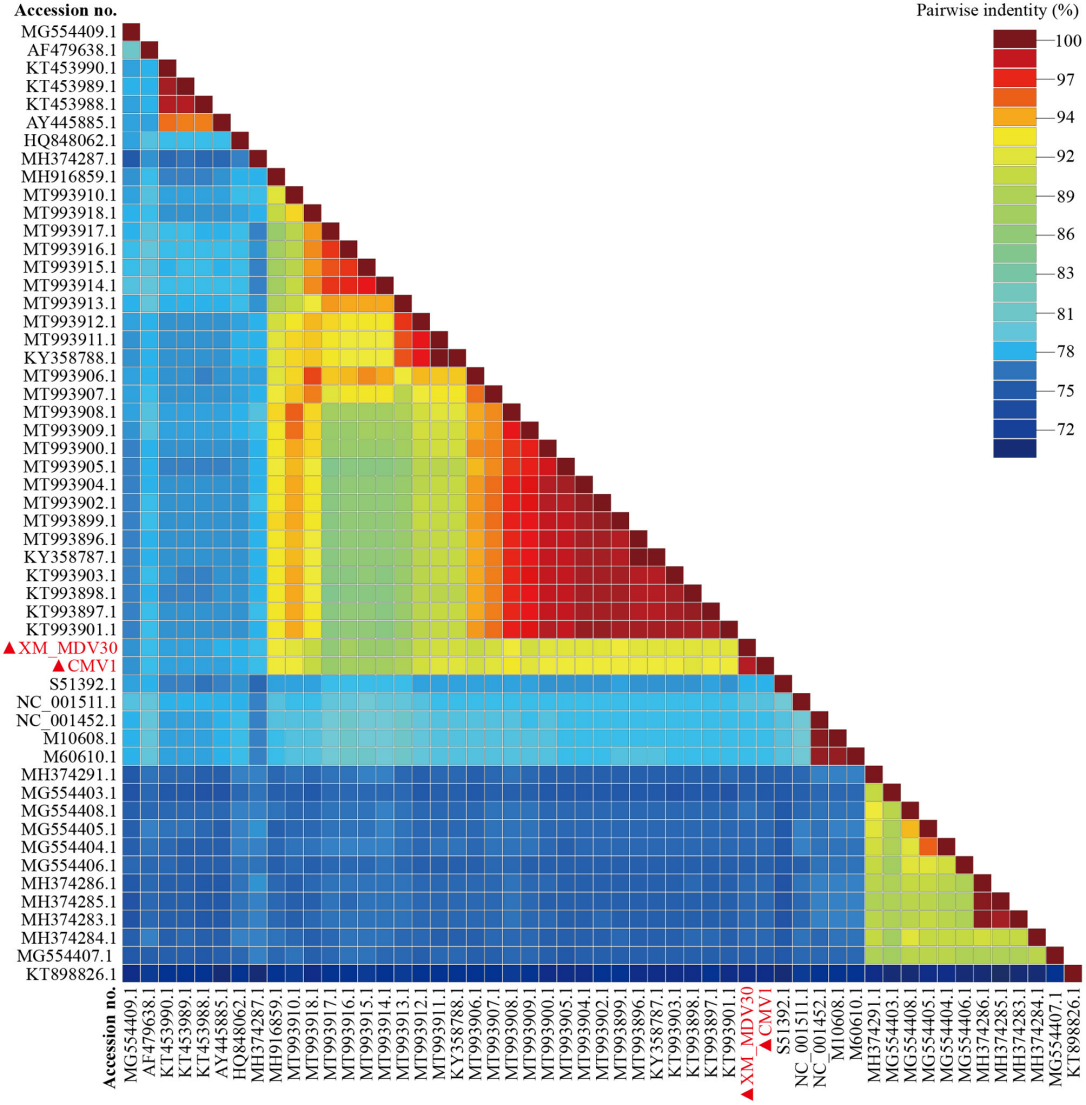
Pairwise whole-genome identity matrix of Visna/Maedi virus strains. The genome sequences only recruited the VMV-like strains (SRLVs genotype A). The VMV genomes sequenced in this study are marked with black triangles.

On comparative analysis, 97.9% identity was observed between CMV-1 and XM-MDV30, and 189 variable sites causing variations were found in three open reading frames (ORFs). The changed ORFs represent three proteins (gag, pol, and env, Supplementary Table S2), with 78.3%−89.1% identity to isolates out of China, with the highest similarity (88.7%−89.1%) with USA strain USMARC-200212120-r, and lowest similarity (78.3%−78.5%) with strain SRLV009 from Italy (Figure 5).

### Phylogenetic Analyses

**Figure 6:**
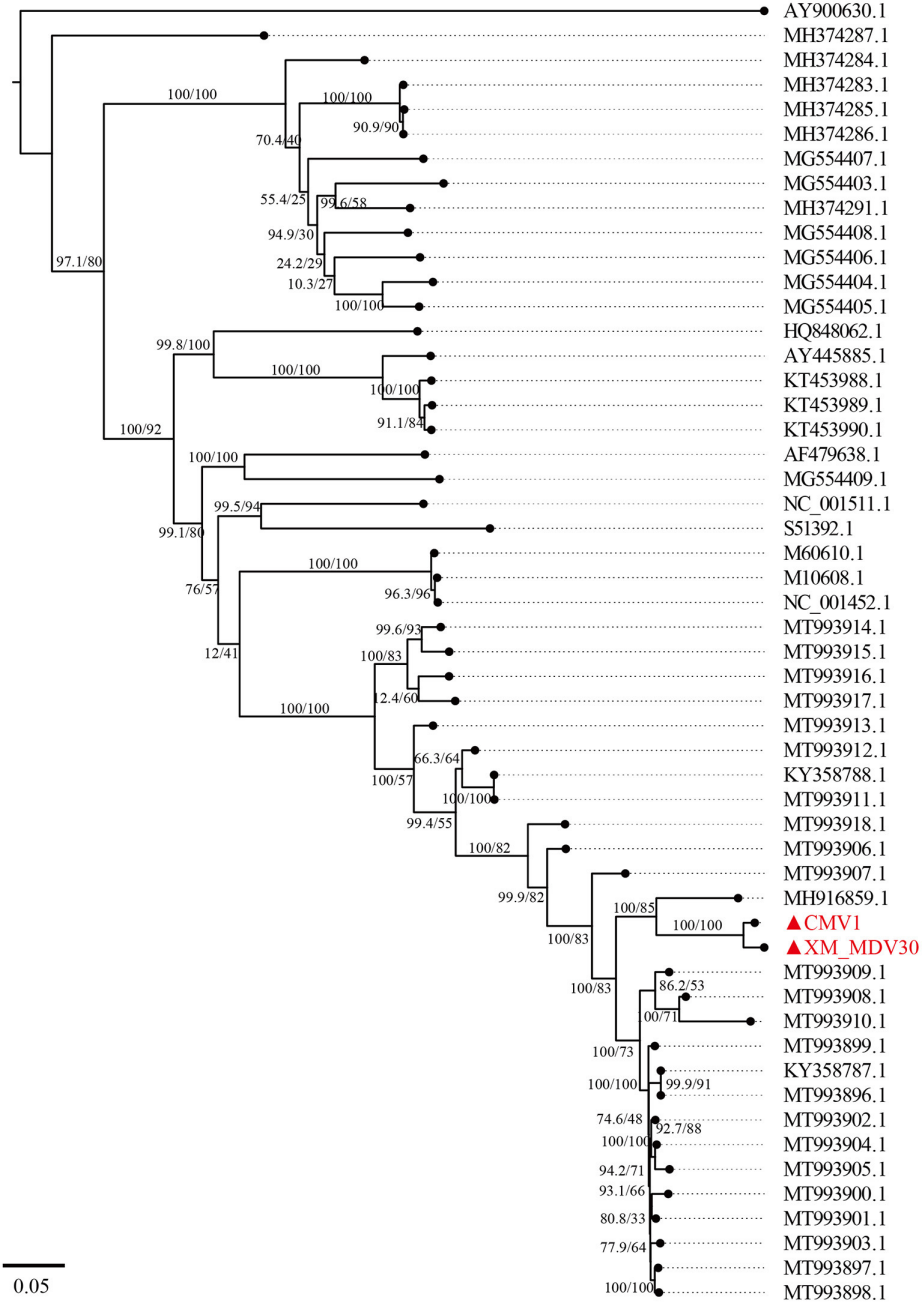
A maximum-likelihood tree of Visna/Maedi virus based on whole-genome sequences. The genome sequences were only recruited the VMV-like strains (SRLVs genotype A). The VMV genomes sequenced in this study are marked with triangles and red color.

A maximum-likelihood tree showed that the two Chinese VMVs are in the same branch as USA strain 1150 (GenBank accession no. MH916859.1; Figure 6). Phylogenetic trees of *env, gag*, and *vif* showed similar results (Supplementary Figures S1, S2, S6), suggesting that the USA and Chinese strains might have a common origin or evolution. In addition, the phylogenetic tree based on encoded genes (*pol, rev*, and *tat*) had different shapes from the tree based on whole-genome sequences (Supplementary Figures S3–S5), suggesting there had been a more complex evolution among VMV strains. Meanwhile, the tree topologies for genome and encoded gene sequences of the Chinese strains were congruent, suggesting that the Chinese strains shared a common ancestor.

## Discussion

Visna/Maedi virus is a high-risk infectious disease in animals in China, and its recessive and latent infections have caused health risks to the national sheep-breeding industry. In this study, two VMV strains isolated from Xinjiang, northwest China in 1985 and 1994 were retrospectively identified using Illumina sequencing and whole-genome cloning. Two precise genome sequences were obtained, which would be beneficial for tracing the source and developing diagnostic methods in China.

A total of 189 variable sites in the genomic sequences caused variations in three ORFs between CMV-1 and XM-MDV30. The altered ORFs encoded three proteins (gag, pol, and env). Of these, pol and env were highly variable, suggesting that these two proteins' diversities probably resulted from adaptive evolutionary pressure. Genomic comparisons of the Chinese VMV strains CMV-1 and XM-MDV30 with those in other countries showed the highest genomic similarity levels (88.7%−89.1%) with the USA strain, with only 78.3%−88.7% identities with other VMV strains. Phylogenetic analyses using whole-genome sequences indicated that CMV-1 and XM-MDV30 were most closely related to USA strain 1150, suggesting that these VMV strains originated from the same ancestor. More circulating VMV strains sequenced in China will help precisely trace the origin.

Visna/Maedi virus spread in sheep and goats worldwide, with about 22 subtypes of VMV genotype A identified and molecularly characterized. A study demonstrated that VMV subtypes were distributed within regions or countries (31), suggesting that the high homologous genetic variation might present in specific regions or countries. Fortunately, only subgenotype A2 was found in the early epidemic period in China, suggesting that the Chinese circulating strain is probably less complicated than those of other countries. However, VMV has been ignored for a long time in China. Currently, limited genomic sequences do not assist in-depth studies that produce eradication programs or diagnostic tests, resulting in challenges in obtaining insights into circulating and genetic diversity in naturally VMV-infected sheep and goats.

Combined with our results showing subgenotype A2 circulated and high seroprevalence presence in China, the findings suggest that implementing control programs would be necessary to block the further spread of VMV. Although current national technical standards and World Organization for Animal Health specifications are implemented to detect and eliminate VMV, limited genomic data remains on the VMV epidemic in China. Therefore, our results would be beneficial for source-tracing and developing a diagnostic method for controlling VMV.

Next-generation sequencing is used to determine the genome sequences of various viruses worldwide (38, 39). Before our study, we failed to directly amplify the whole-genome sequences using several primers referred to as the reference genome sequence in GenBank before combining Illumina sequencing and whole-genome cloning methods, which might be attributed to a low level of similarity between the Chinese VMV strains and strains outside of China. This situation suggests that next-generation sequencing technologies help determine the sequences of highly variable viruses.

## Conclusions

Illumina sequencing and whole-genome cloning confirmed that the two Chinese VMV strains (CMV-1 and XM-MDV30) belong to subgenotype A2 and share 78.3%−89.1% identities with other strains. The two Chinese VMV strains were highly homologous, and were genetically related to USA strain 1150. This study's conclusions apply to carrying out epidemiological investigations and implementing control strategies. A comprehensive molecular epidemiological investigation is warranted to reveal the molecular characterization and viral evolution in Chinese VMV isolates in the future.

## Data Availability Statement

The datasets presented in this study can be found in online repositories. The names of the repository/repositories and accession number(s) can be found in the article/Supplementary Material.

## Author Contributions

J-YW and Y-RW conceived and designed the experiments and finalized the manuscript. J-YW, X-YM, X-YY, JW, X-XM, HB, and Y-RW performed the experiments and analyzed the data. J-YW drafted the manuscript. All authors read and approved the contents of the manuscript.

## Funding

This work was funded by the National Key Research and Development Program of China (2016YFD0500908) and the Xinjiang Project for Construction of Veterinary Microbiological Resources and Sharing Platform (PT1809).

## Conflict of Interest

The authors declare that the research was conducted in the absence of any commercial or financial relationships that could be construed as a potential conflict of interest.

## Publisher's Note

All claims expressed in this article are solely those of the authors and do not necessarily represent those of their affiliated organizations, or those of the publisher, the editors and the reviewers. Any product that may be evaluated in this article, or claim that may be made by its manufacturer, is not guaranteed or endorsed by the publisher.

## Supplementary Material

Supplementary Figure S1 A maximum-likelihood tree of Visna/Maedi virus based on the *env* gene. The *env* sequences were only recruited the VMV-like strains (SRLVs genotype A). The VMV genomes sequenced in this study are marked with triangles and red color.

Supplementary Figure S2 A maximum-likelihood tree of Visna/Maedi virus based on the *gag* gene. The *gag* sequences were only recruited the VMV-like strains (SRLVs genotype A). The VMV genomes sequenced in this study are marked with triangles and red color.

Supplementary Figure S3 A maximum-likelihood tree of Visna/Maedi virus based on the *pol* gene. The *pol* sequences were only recruited the VMV-like strains (SRLVs genotype A). The VMV genomes sequenced in this study are marked with triangles and red color.

Supplementary Figure S4 A maximum-likelihood tree of Visna/Maedi virus based on the *rev* gene. The *rev* sequences were only recruited the VMV-like strains (SRLVs genotype A). The VMV genomes sequenced in this study are marked with triangles and red color.

Supplementary Figure S5 A maximum-likelihood tree of Visna/Maedi virus based on the *tat* gene. The *tat* sequences were only recruited the VMV-like strains (SRLVs genotype A). The VMV genomes sequenced in this study are marked with triangles and red color.

Supplementary Figure S6 A maximum-likelihood tree of Visna/Maedi virus based on the *vif* gene. The *vif* sequences were only recruited the VMV-like strains (SRLVs genotype A). The VMV genomes sequenced in this study are marked with triangles and red color.

Supplementary Table S1 Data for Illumina sequencing for the two Chinese Visna/Maedi virus strains.

Supplementary Table S2 Mutation sites of Amino acid between Chines Visna/Maedi virus isolates.
