# Supplementary figures and images for: The First Genomic Analysis of Visna/Maedi Virus Isolates in China
Source: Front Vet Sci. 2022 Jun 24;9:846634. doi: 10.3389/fvets.2022.846634 (PMC9263623; doi:10.3389/fvets.2022.846634)

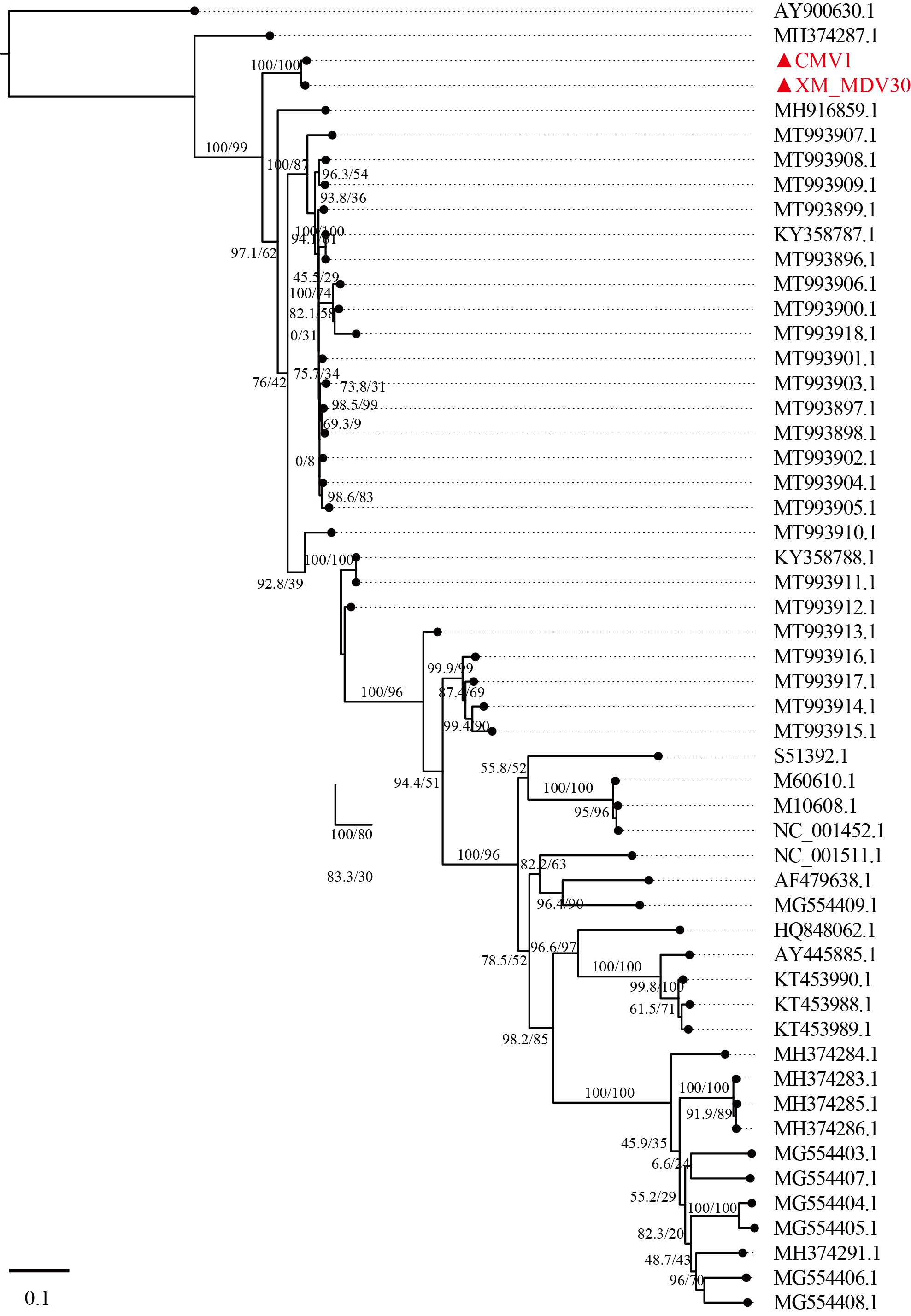

Supplement: Supplementary Figure S1 — A maximum-likelihood tree of Visna/Maedi virus based on the env gene. The env sequences were only recruited the VMV-like strains (SRLVs genotype A). The VMV genomes sequenced in this study are marked with triangles and red color. [file Image_1.TIF]

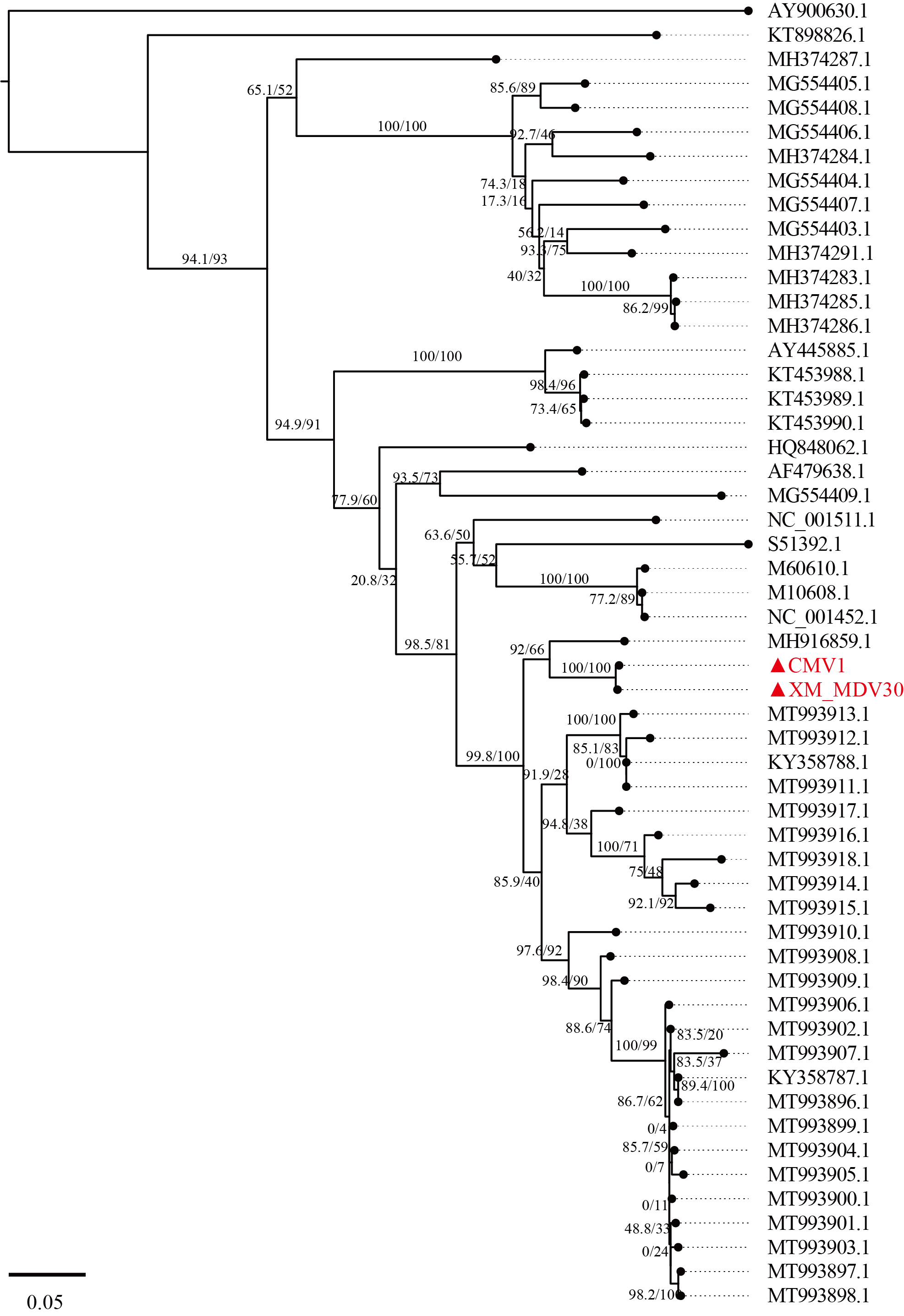

Supplement: Supplementary Figure S2 — A maximum-likelihood tree of Visna/Maedi virus based on the gag gene. The gag sequences were only recruited the VMV-like strains (SRLVs genotype A). The VMV genomes sequenced in this study are marked with triangles and red color. [file Image_2.TIF]

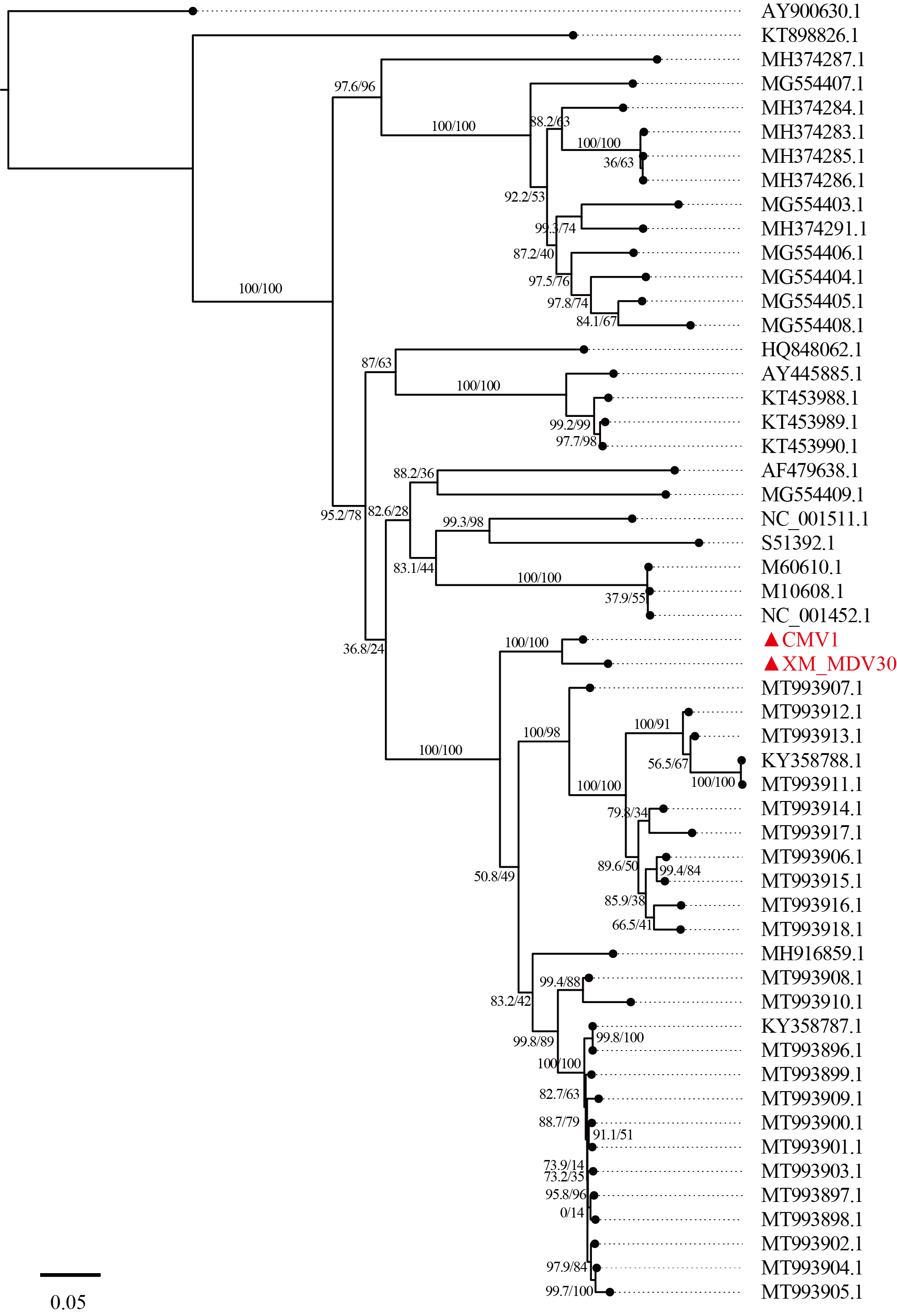

Supplement: Supplementary Figure S3 — A maximum-likelihood tree of Visna/Maedi virus based on the pol gene. The pol sequences were only recruited the VMV-like strains (SRLVs genotype A). The VMV genomes sequenced in this study are marked with triangles and red color. [file Image_3.TIF]

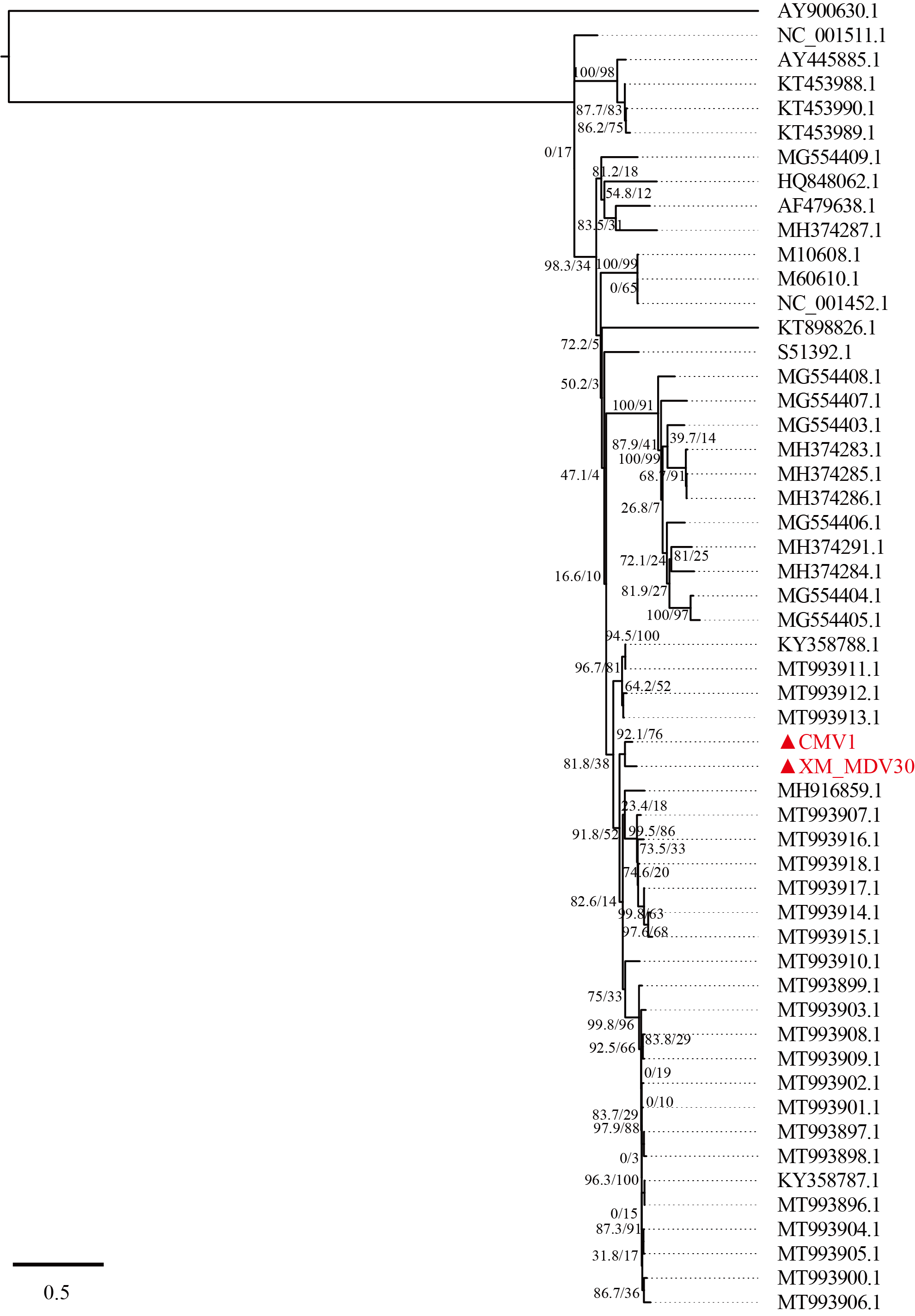

Supplement: Supplementary Figure S4 — A maximum-likelihood tree of Visna/Maedi virus based on the rev gene. The rev sequences were only recruited the VMV-like strains (SRLVs genotype A). The VMV genomes sequenced in this study are marked with triangles and red color. [file Image_4.TIF]

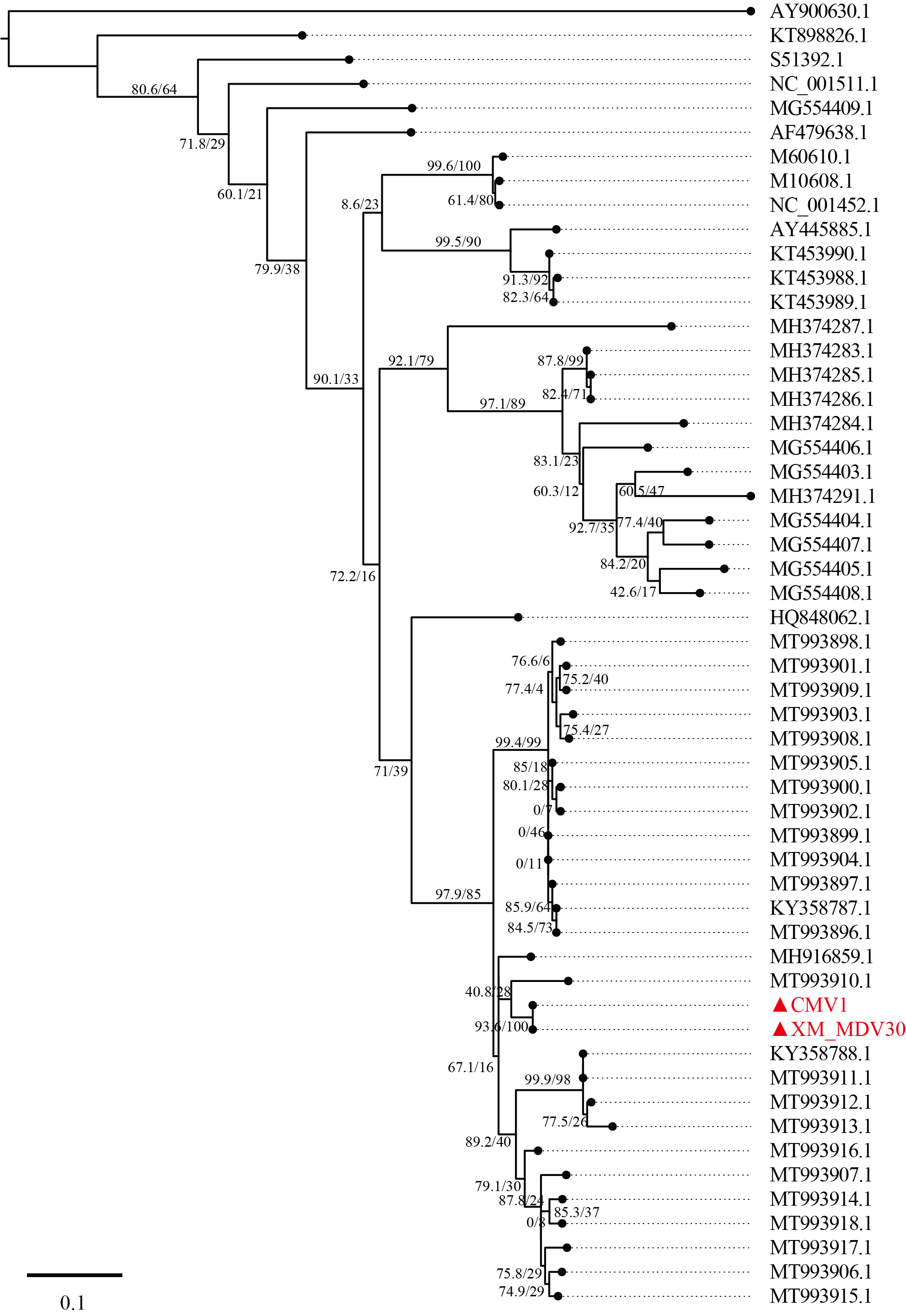

Supplement: Supplementary Figure S5 — A maximum-likelihood tree of Visna/Maedi virus based on the tat gene. The tat sequences were only recruited the VMV-like strains (SRLVs genotype A). The VMV genomes sequenced in this study are marked with triangles and red color. [file Image_5.TIF]

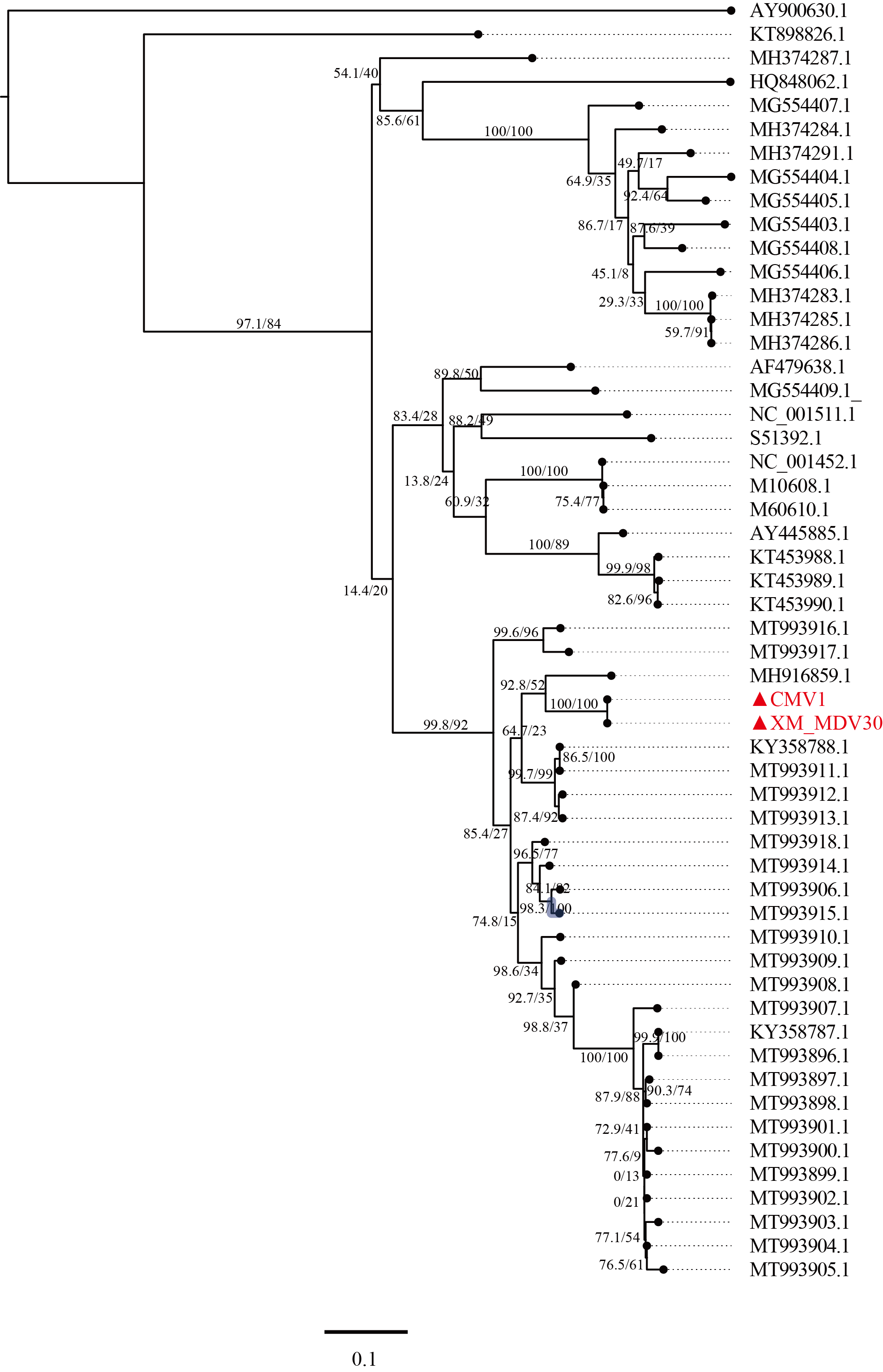

Supplement: Supplementary Figure S6 — A maximum-likelihood tree of Visna/Maedi virus based on the vif gene. The vif sequences were only recruited the VMV-like strains (SRLVs genotype A). The VMV genomes sequenced in this study are marked with triangles and red color. [file Image_6.TIF]
