# Supplementary material for: The First Genomic Analysis of Visna/Maedi Virus Isolates in China
Source: Front Vet Sci. 2022 Jun 24;9:846634. doi: 10.3389/fvets.2022.846634 (PMC9263623; doi:10.3389/fvets.2022.846634)
Supplement: Supplementary Table S1 — Data for Illumina sequencing for the two Chinese Visna/Maedi virus strains. [file Table_1.docx]

## Table S1. Data for Illumina sequencing for the two Chinese Visna/Maedi virus isolates

| **Sample ID** | **Raw Data (Mb)** | **Clean Data (Mb)** | **Clean Data (%)** |
| --- | --- | --- | --- |
| CMV-1 | 2,847 | 2,518 | 88.4 |
| XM-MDV30 | 3,252 | 2,835 | 87.2 |
