# Supplementary material for: The First Genomic Analysis of Visna/Maedi Virus Isolates in China
Source: Front Vet Sci. 2022 Jun 24;9:846634. doi: 10.3389/fvets.2022.846634 (PMC9263623; doi:10.3389/fvets.2022.846634)
Supplement: Supplementary Table S2 — Mutation sites of Amino acid between Chines Visna/Maedi virus isolates. [file Table_2.docx]

**Table S2. Mutation sites of amino acid between Chines Visna/Maedi virus isolates**

| **Protein** | **Mutation position** | XM-MDV30 | CMV-1 |
| --- | --- | --- | --- |
| gag | 24 | R | V |
|  | 223 | V | I |
|  | 237 | Q | H |
|  | 422 | R | S |
| pol | 49 | M | I |
|  | 103 | P | Q |
|  | 108 | N | E |
|  | 110 | I | T |
|  | 112 | K | R |
|  | 120 | S | T |
|  | 132 | G | A |
|  | 134 | I | L |
|  | 139 | V | I |
|  | 142 | T | N |
|  | 146 | R | K |
|  | 152 | E | Q |
|  | 154 | R | K |
|  | 163 | N | H |
|  | 182 | D | N |
|  | 243 | Q | K |
|  | 244 | K | R |
|  | 247 | H | N |
|  | 338 | T | K |
|  | 342 | K | E |
|  | 345 | E | N |
|  | 349 | N | S |
|  | 989 | I | L |
| vif | — |  |  |
| tat | — |  |  |
| Env | 126 | A | V |
|  | 321 | I | V |
|  | 432 | N | K |
|  | 436 | Y | H |
|  | 573 | R | K |
|  | 576 | K | I |
|  | 793 | L | V |
|  | 881 | D | G |
|  | 917 | I | M |
|  | 919 | M | T |
|  | 927 | S | G |
|  | 928 | I | D |
|  | 929 | G | K |
|  | 931 | R | K |
|  | 932 | R | K |
|  | 936 | V | M |
|  | 941 | V | I |
|  | 942 | I | L |
|  | 943 | V | I |
|  | 944 | M | I |
|  | 950 | E | A |
|  | 954 | T | I |
|  | 957 | S | N |
|  | 960 | R | K |
|  | 961 | N | K |
|  | 982 | V | A |
|  | 986 | INDEL | K |
|  | 987 | INDEL | Q |
|  | 988 | INDEL | L |
| Rev |  |  |  |
